# Toward burnout prevention with Bayesian mixed-effects regression analysis of longitudinal data from wearables: a preliminary study

**DOI:** 10.3389/fdgth.2025.1640900

**Published:** 2025-08-28

**Authors:** Radoslava Švihrová, Davide Marzorati, Michal Bechný, Max Grossenbacher, Yuriy Ilchenko, Jürg Grossenbacher, Athina Tzovara, Francesca Dalia Faraci

**Affiliations:** ^1^ Institute of Computer Science, Faculty of Science, University of Bern, Bern, Switzerland; ^2^ Department of Innovative Technologies, Institute of Digital Technologies for Personalized Healthcare, University of Applied Sciences and Arts of Southern Switzerland, Lugano, Switzerland; ^3^ Resilient SA, Lausanne, Switzerland; ^4^ Psy Bern AG, Bern, Switzerland; ^5^ Center for Experimental Neurology, Department of Neurology, Inselspital, Bern University Hospital and University of Bern, Bern, Switzerland

**Keywords:** Bayesian analysis, burnout, mixed model, sleep, stress, wearables

## Abstract

Wearable devices have gained significant popularity in recent years, as they provide valuable insights into behavioral patterns and enable unobtrusive continuous monitoring. This work explores how daily lifestyle choices and physiological factors contribute to coping capacities and aims at designing burnout prevention systems. Key variables examined include sleep stage proportions and nocturnal stress levels, as both play a crucial role in recovery and resilience. Longitudinal data from a 1-week study incorporating wearable-derived features and contextual information are analyzed using a mixed-effects model, accounting for both overall trends and individual differences. A Bayesian inference approach is exploited to quantify uncertainty in estimated effects, providing their probabilistic interpretation and ensuring robustness despite the low sample size. Findings indicate that alcohol consumption negatively affects rapid-eye-movement sleep, increases awake time, and elevates nocturnal stress. Excessive daily stress reduces deep sleep, while an increase in daily active hours promote it. These results align with the existing literature, demonstrating the potential of consumer-grade wearables to monitor clinically relevant relationships and guide interventions for stress reduction and burnout prevention.

## 1 Introduction

The World Health Organization (WHO) defines burnout as a syndrome resulting from chronic workplace stress that has not been successfully managed (1). A recent US employee well-being survey (1,200 employers and 2,001 employees) found that over 59% of American workers experience moderate levels of burnout (2). Furthermore, among those affected, 86% reported additional mental health issues in the previous 12 months, including anxiety, depression, and sleep disturbances (2). According to recent statistics, burnout syndrome is even more prevalent among health workers, a situation that was exacerbated by the global COVID-19 pandemic, which further increased its already high prevalence (3, 4).

Burnout carries substantial economic implications, including an estimated $4.6 billion in annual US healthcare expenditures attributed to its effects, such as increased physician turnover and reduced clinical work hours (5). Given that burnout continues to affect a growing workforce and contributes to rising economic losses, early risk identification and effective prevention strategies are increasingly needed. Burnout is typically assessed using validated psychometric clinical scales that evaluate workspace well-being and work-related stress, such as the Maslach Burnout Inventory (MBI) (6) or the Shirom-Melamed Burnout Measure (SMBM) (7, 8). While these tools remain essential, recent research has started to incorporate multimodal data to capture burnout risk more dynamically. For example, ecological momentary assessment has been used to monitor real-time affect and fatigue in workplace settings (9), while wearable-based physiological measures, including heart rate variability (HRV) and sleep stage tracking, have been explored as objective markers of chronic stress and burnout (10, 11). Several authors compared wearable-based data to those provided by medical-grade devices. For example, Kainec et al. (12) reported that consumer-grade sleep tracking devices are a cost-effective and accurate solution for sleep measurement. Other researchers have also identified Garmin smartwatches as accurate and sensitive in detecting steps (13) and monitoring sleep (14). Furthermore, several studies have evaluated the accuracy of photoplethysmography (PPG) sensors; for example, some have focused on for telemonitoring (15) and others on comparisons to electrocardiograms (16). A meta-analysis (17) concluded that PPG-based wearable devices show acceptable validity for heart-rate measurements. It is worth noting that while these devices are not yet fully comparable to medical-grade devices for detecting sleep stages (14, 18, 19), the insights they provide into individual-level trajectories can provide unprecedented opportunities in long-term remote monitoring (20). This highlights the high priority of understanding and implementing preventive strategies through interventions that target both individual behaviors and broader structural factors at the population level (21).

Sleep, as one of the core lifestyle pillars, is deeply connected to mental health and stress regulation. Consequently, interventions and observational studies focusing on sleep are crucial for understanding and mitigating burnout risk (22). Several studies have reported that sleep issues can both contribute to or be affected by burnout (23–25). A decline in sleep quality can be considered an early marker of chronic stress and upcoming burnout (26). In the presence of burnout, sleep becomes more fragmented, with frequent awakenings reducing sleep efficiency (27). This leads to an increase in non-restorative light (N1/N2) sleep at the expense of deep sleep (N3), which is essential for physical restoration, and rapid-eye-movement (REM) sleep, which is crucial for cognitive restoration (28). Sleep disruptions not only contribute to fatigue (29) but can also impair coping capacity, while high-quality, consolidated sleep is associated with greater resilience and improved emotional regulation. Given the central role of sleep in mental health and burnout prevention, a better understanding of the interplay between physiological recovery processes, daily behaviors, and stress coping mechanisms is essential.

Wearable devices and mobile applications for health monitoring have become ubiquitous in everyday live and clinical monitoring (30). Aggregated daily data from wearable sensors, comprising multimodal signals such as heart rate (HR), HR variability (HRV), activity, and sleep, can provide valuable insights into personalized daily behavioral patterns (31). These signals can serve as physiological proxies for perceived comfort and affective states, offering promising opportunities for the continuous and unobtrusive assessment of personal well-being (32). To further enhance these physiological insights, the data can be combined with implicit contextual information from background phone information (e.g., location, calendar date,…) and explicit context collected via questionnaires (e.g., alcohol intake, working hours,…). Several recent studies have explored the effects of daily lifestyle choices on sleep physiology. For example, alcohol intake was shown to affect sleep quality using random intercept models (33), while its impact on nocturnal resting heart rate was assessed in a similar study (34). In addition, Bustamante et al. (35) investigated the interaction between perceived stress and actigraphy-derived sleep metrics in students, comparing individual-level and time-derived features. In this study, we aim to evaluate associations between lifestyle behaviors and sleep-related physiological metrics by employing a Bayesian mixed-effects model. This approach is ideal for accounting for individual variability in longitudinal data collected over consecutive days, as it can captures both general trends and within-subject variations to quantify how lifestyle factors interact with sleep and stress. Moreover, compared to the classical frequentist approach, posterior distributions over the estimated parameters can be explored, which is favorable especially in the case of small sample sizes. While prior studies have used mixed models for specific behavioral effects, our method offers a more comprehensive view of physiological coping by integrating subjective and objective daily-level predictors with sleep macroarchitecture and autonomic stress responses.

In this study, we assess *coping capacity* through two key physiological markers: (1) the balance of interdependent sleep stage proportions, which represents sleep macroarchitecture, and (2) nocturnal stress levels, which reflects autonomic nervous system activation during sleep. By quantifying the impact of lifestyle factors on these markers, we aim to enhance our understanding of the physiological and behavioral determinants of coping capacity. The findings can inform future studies aiming to analyze causal lifestyle effects and guide the development of resilience-building strategies, such as encouraging behaviors that promote restorative sleep.

## 2 Materials and methods

In this section, we describe the collection of wearable-derived and self-reported contextual data. In addition, we outline the derivation of key sleep and stress markers and detail the statistical approach used to assess lifestyle impacts through longitudinal data.

### 2.1 Study data

#### 2.1.1 Participants and protocols

Data were collected over a 1-week period in August 2021 from 22 academic workers recruited by email from institutes at the University of Applied Sciences and Arts of Southern Switzerland. At the beginning of data collection, study participants completed a digital onboarding questionnaire focused on sociodemographics characteristics (age, sex, weight, career level) and health-related factors (medication, smoking status). Along with the onboarding questionnaire, the SMBM questionnaire was administered. For the duration of the week, participants were asked to continuously wear a Garmin Venu SQ smartwatch and complete brief daily questionnaires: once in the morning after waking up and again in the evening before going to sleep. Informed written consent was obtained from all participants, and a “Clarification of responsibility” from the cantonal ethics committees (swissethics) revealed that no ethical approval was needed for this study (Req-2021-00451). The study was conducted according to Helsinki guidelines for ethical research (36). After data collection, all data were stored securely on secure servers accessible only to authorized researchers. Data were stored in a coded format, with a random-generated code assigned to each participant. Participants’ personal and sensitive information was stored separately and was accessible only to the principal investigator of the study (FF).

#### 2.1.2 Wearable data

Physiological and activity data were accessed via the Garmin Health API.^1^ The Garmin smartwatch exploits optical sensors to measure blood volume pulse (BVP), from which heart rate (HR), heart rate variability (HRV), and respiration rate are derived. Moreover, Garmin’s proprietary algorithms (37) compute stress levels and body battery (an energy metric) on a scale from 0 to 100, mostly based on HRV through a blackbox algorithm. If the device is worn at night, sleep is recorded and automatically detects sleep stages with a 1 min resolution. Garmin provides a different terminology for sleep stages compared to the standard nomenclature: *deep* sleep refers to the N3 stage, while *light* sleep is composed of both N1 and N2 stages. Additional metrics include step count, tracked sports activities, and 15-min epoch summaries of sedentary, active, and highly active time.

#### 2.1.3 Derived markers

The wearable data were summarized into longitudinal daily features, describing individuals’ physiological characteristics of sleep and stress.

**Sleep variables** captured sleep architecture as proportions of time spent in different sleep stages (i.e., awake, light, deep, REM). In addition, the total sleep duration was recorded, and sleep–wake cycles were identified based on the first asleep and final wake-up times.

**Stress characteristics** were based on Garmin-provided stress levels, originally reported on a 0–100 scale. For each sleep–wake cycle, summary statistics (mean, standard deviation, minimum, maximum, and median) were calculated separately for awake and asleep periods. Stress levels were further categorized into four equidistant classes, as in the user interface of the associated Garmin mobile application, and the proportion of time spent in each category was computed for both sleep and wake periods.

#### 2.1.4 Self-reported and contextual data

Because physiological data alone do not fully capture an individual’s psychological state, subjective and contextual information are essential. This study distinguishes between *implicit* (derived) and *explicit* (self-reported) information. Implicit data included weekday vs. weekend classification based on wearable timestamps. Explicit context was gathered via self-reported morning and evening questionnaires, where participants rated their mood, stress, and energy on a 10-point Likert scale. The evening questionnaire also recorded alcohol intake (none, low, moderate, high), perceived morning and evening work commute, workplace crowdedness, and total hours worked.

### 2.2 Study outcomes

Similar to Bechný et al. (38) and in collaboration with a clinical professional, we defined two key physiological outcomes to assess individual’s coping capacity markers, which are assumed to be time-varying mediators of burnout, and we examined how they are associated with behavioral patterns and lifestyle choices. Specifically, by leveraging wearable and self-reported data, we investigated how daily habits, daytime stress, and contextual factors influence sleep and nocturnal stress profiles, which we consider as two proxies for measuring coping capacity.

1. *Sleep profile*: We examined how different lifestyle factors contribute to variations in deep and REM sleep proportions, assuming these to be linked with physical and cognitive regeneration, respectively. Moreover, high proportions of awakenings during the night indicate lower sleep quality and thus lower regeneration.
2. *Nocturnal stress profile*: Similarly, we quantified how lifestyle factors relate to the proportion of time spent in individual nocturnal stress levels. Higher proportions of high-stress levels during the night indicate lower sleep quality and thus lower regenerative and coping capability.

By modeling these outcomes, we evaluated which lifestyle factors (e.g., alcohol intake, daily stress, and active hours) disrupt or promote recovery and assessed their impact on nighttime stress levels. These insights will contribute to the design of future personalized interventions aimed at enhancing sleep quality, stress resilience, and burnout prevention.

### 2.3 Statistical methods

Both outcomes are compositional data, meaning that their interdependent components sum up to 1; i.e., they are points on a simplex (39). To appropriately model these D-dimensional outcomes, we applied the additive log-ratio (ALR) transformation (39), defined as follows:

(1) $$\text{alr}(p)=\left[\ln\frac{\;p_{1}}{p_{D}},\ldots ,\ln\frac{\;p_{D-1}}{p_{D}}\right]$$

where $p_{D}$ serves as the baseline component, enabling log-ratio comparisons of the original vector of proportions p. This transformation reduces dimensionality by one and, thanks to the logarithm, ensures a near-normal distribution, desirable for the regression analysis.

To model multidimensional longitudinal outcomes Y, we employed a multiple multivariate mixed-effects model with individual-specific random intercepts (40):

(2) Y=Xβ+Zu+ϵ

where X and Z are design matrices, β and u represent fixed and random regression coefficients, respectively, and Y consists of (D−1) ALR-transformed outcome components. While β provides estimates of overall (population) effects, the random intercepts account for repeated within-subject measurements and thus correctly handle the repeated measurement data.

Regression parameters were estimated in a Bayesian manner (41). The prior distributions for the model parameters were specified using the normal distribution and utilizing conjugate priors. Posterior inference was carried out using Markov chain Monte Carlo (MCMC) sampling with five Markov chains, each with 5,000 iterations (including warm-up, for which half of the iterations were used), and random initialization. For sampling, a No-U-Turn algorithm was used (42). This enabled the computation of credibility intervals and the derivation of the *probabilities of direction* (PoDs) (41), which quantify the proportion of the posterior distribution sharing the same sign as the (median) effect estimates, aiding interpretation of effects and assessing the direction of their association (43). This analysis was performed using the statistical software R (44) and the Stan package for R (45), with default prior specifications.

## 3 Results

The proposed framework of multiple multivariate mixed-effects models for longitudinal compositional outcomes from Equations 1 and 2 was used to evaluate two research questions, assessing the impact of daily lifestyle factors and physiological values on *sleep profile* and *noctural stress profile*, respectively.

### 3.1 Data preparation

Data from six participants were excluded due to incomplete sleep data collected during the 1-week study. Therefore, we used data only from 16 participants (including 14 men and 2 women). Age ranged from 24 to 44 years, with a mean (SD) of 34 (5.92). SMBM scores ranged from 1.46 to 5.46, with a mean (SD) of 2.9 (1.14), out of a maximal score of 7 (where lower scores indicate a lower risk of burnout). Given the sample size and the absence of participants diagnosed with burnout, we could not properly evaluate the relationship between SMBM and the two defined coping capacity markers in a data-driven way.

Among the included participants, 11 completed all morning and evening questionnaires, while the remaining had up to 3 missing answers. These missing data points were mean-imputed, conditioning on individuals and weekend indicators.

To mitigate multicollinearity, a subset of relevant variables was selected while accounting for highly correlated predictors. Given the limited diversity in gender (only two women) and smoking status (only two daily smokers), these variables were not considered as predictors. Similarly, career level was omitted due to its direct correlation with participants’ age (cor = 0.79). Self-reported assessments conducted in the morning were also excluded due to the considerable time gap between these measures and night-related variables. Among the evening self-reported assessments, subjective energy and stress levels were retained as predictors, being timely close to the night-measured outcomes. The amount of daily work hours was considered among the predictors, providing context regarding participants’ workload. To examine the effect of (physical) activity time on the outcomes, we incorporated the sum of active and highly active hours as a single predictor, whereas daily steps were removed due to their high correlation with activity time (cor = 0.82). In addition, due to a limited number of responses for high alcohol intake, it was merged into the moderate category, while low intake remained separate. These categories were then one-hot encoded as distinct predictors to differentiate between individual consumption levels. To enhance the interpretation of the estimated effects, we centered age and average daily stress at a value of 30, ensuring that the model’s intercept reflects the expected values for individuals at this reference level.

### 3.2 Effects of lifestyle on sleep profile

To assess the associations between different lifestyle choices, behaviors and daily stress with sleep, we first used the sleep profile as an output for the regression model. To prevent zeros in relative (ALR) comparisons, Laplace smoothing (λ=0.01) was applied. The ALR transformation from Equation 1 was applied with the light sleep stage proportion as the baseline. This yielded three ALR-transformed outcomes (for deep sleep, REM sleep, and wakefulness to light sleep), which were further modeled by the proposed mixed-effects model with a random intercept, as specified in Equation 2.

Table 1 reports the posterior medians as the estimated effects (EEs) and PoDs on the sleep stage proportions. To draw a parallel to hypothesis testing in a Bayesian setting, we highlight all PoD values > 0.75 (except for the intercept) in Table 1, as these values correspond to the Bayes factor >3, which is considered substantial evidence (46). This means that at least 3/4 of the posterior samples had the same sign (i.e., the direction of the association) as the EE.

**Table 1:** Estimated median fixed effects on sleep stage proportions.

Coefficient	ALR outcome					
	$\ln\frac{\%\mathrm{Deep}}{\%\mathrm{Light}}$		$\ln\frac{\%\mathrm{REM}}{\%\mathrm{Light}}$		$\ln\frac{\%\mathrm{Awake}}{\%\mathrm{Light}}$	
	EE	PoD	EE	PoD	EE	PoD
(Intercept)	−1.97	1.00	−1.39	1.00	−4.44	1.00
Age-30	−0.03	**0.85**	0.02	**0.76**	0.00	0.62
Low alcohol intake	0.37	**0.94**	−0.19	**0.86**	0.04	0.61
Moderate alcohol intake	0.29	**0.86**	−0.55	**1.00**	−0.16	**0.81**
Hours worked	0.01	0.62	0.02	**0.85**	0.00	0.60
Subjective evening stress	0.00	0.50	0.04	**0.79**	−0.00	0.52
Subjective evening energy	−0.02	0.60	−0.01	0.56	0.04	**0.83**
Active hours	0.12	**0.92**	0.03	0.66	0.00	0.52
Average daily stress-30	−0.03	**1.00**	0.01	**0.81**	−0.00	0.54

EE, estimated effect; PoD, probability of direction. PoD > 0.75, corresponding to Bayes factor > 3 are highlighted in bold.

Consistent with clinical observations (47), the deep-to-light ratio decreased (PoD = 0.85) with increasing age. Both low and moderate alcohol consumption levels were associated with an increase in deep sleep (PoD of 0.94–0.86). A similar positive association (PoD = 0.92) was observed for active hours. This suggests that, as highlighted in McCullar et al. (48), alcohol may initially promote sleep by increasing the occurrence of deep sleep. However, alcohol intake ultimately impairs REM sleep and overall sleep quality (49). Physical activity was also found to facilitate deeper restorative states and support physiological recovery, in agreement with (50–52).

**Figure 1:**
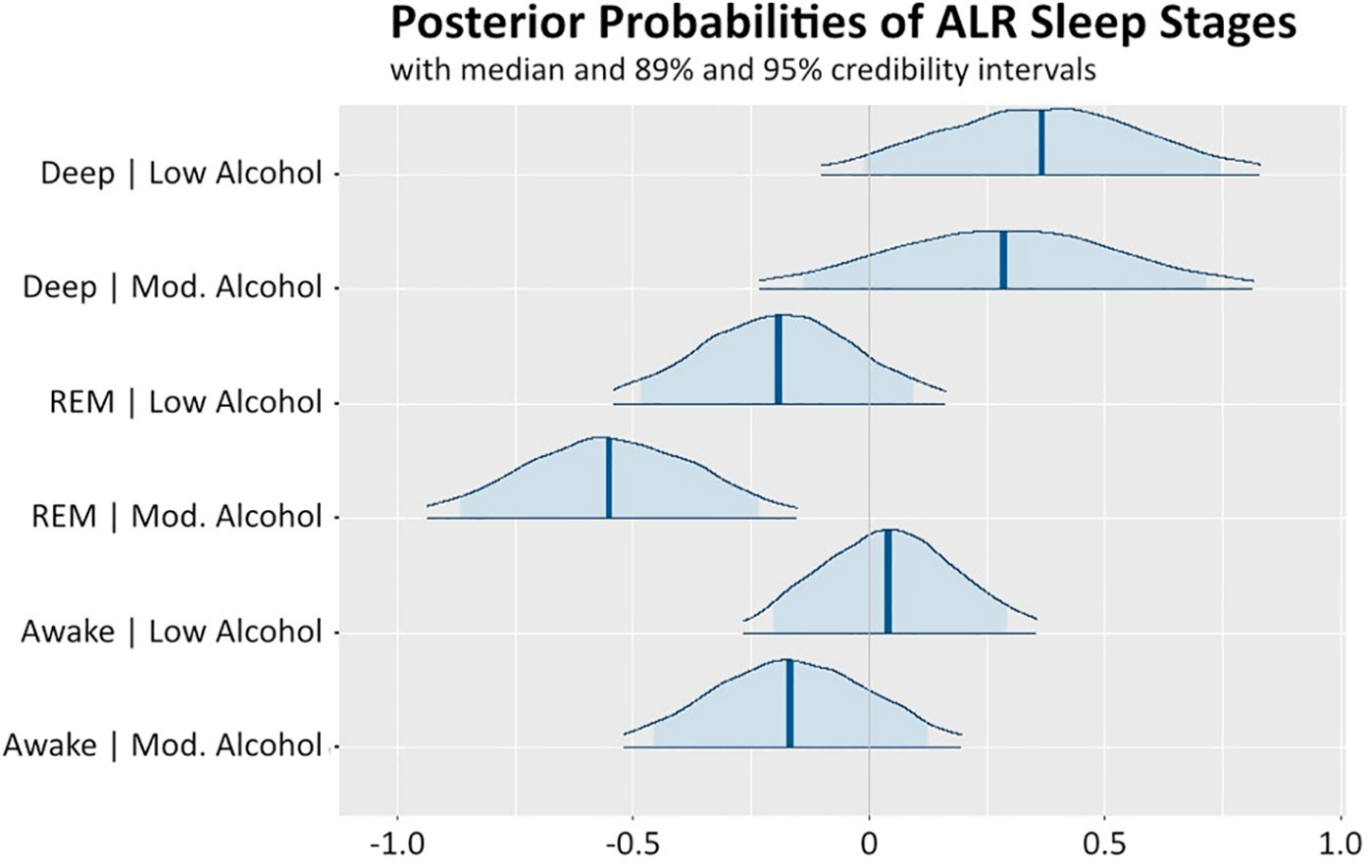
Posterior distributions of effects of low and moderate alcohol intake on the three ALR-transformed sleep stages (deep, REM, awake to light sleep). The highlighted vertical lines in the posterior probability distributions represent the medians, dark blue lines represent the 95% credibility intervals, and the light blue highlighted areas correspond to the 89% credibility intervals. Values on the *x*-axis represent the magnitude.

In our study, the observed REM increase with age (PoD = 0.76), which is rather uncommon in literature (47), may reflect job seniority (cor = 0.79 with age), which is linked to higher cognitive workload and hence REM pressure. In contrast to deep sleep, even a low alcohol intake negatively impacts (PoD = 0.86) REM sleep. The EE was almost three times greater for moderate alcohol intake (−0.55 vs. −0.19 for moderate vs. low alcohol intake, respectively), whose effect was found negative with high credibility (PoD = 1). The influence of alcohol intake on the REM-to-light sleep ratio is elaborated in detail in Figure 1. This finding aligns with existing research indicating that alcohol consumption alternates and disrupts REM sleep (53, 54). Further, all other predictors (amount of daily work hours, subjective evening stress, and average daily stress) were associated with increased REM, although their effects were very small (0.02, 0.04, 0.01), with PoD values ranging from 0.79 to 0.81. As REM is responsible for processing cognitive and emotional workload, this might suggest an increased need for coping with these demands in healthy individuals. It is important to note, however, that chronic stress and increased workload may lead to decreased REM, which negatively impacts memory consolidation and emotional regulation (55). The awake-to-light sleep ratio was positively (PoD = 0.83) influenced by subjective evening energy, leading to decreased sleep efficiency. Conversely, moderate alcohol intake had a negative impact (PoD = 0.81), suggesting that moderate alcohol intake leads to fewer awakenings or that it leads to a more pronounced increase in light sleep. Estimated sigmas, quantifying individual-level variability in the random intercepts, in modeling the three ALR-transformed outcomes (deep sleep, REM sleep, and wakefulness to light sleep), were 0.88, 0.68, and 0.65, respectively. Visual inspection of residual plots did not reveal any violations of homoscedasticity or normality assumptions.

### 3.3 Effects of lifestyle on noctural stress profile

Similarly, we further assessed the impact of different behaviors, lifestyles, and daily stress on the nocturnal stress profile. Due to a limited number of observations in the highest stress level (76–100) during the night and to overcome numerical instability, we merged the two higher categories, resulting in three stress level proportions: low (0–25), medium (26–50), and high (51–100). The ALR-transformation from Equation 1 was applied using the low-stress category as the baseline. The model from Equation 2 was then jointly estimated on both ALR-transformed components. The posterior medians of the estimated effects (EEs) on the proportions of nocturnal stress, along with the corresponding PoDs, are reported in Table 2. PoD values > 0.75, corresponding to the substantial evidence, are highlighted in bold.

**Table 2:** Estimated median fixed effects on nocturnal stress proportions.

Coefficient	ALR outcome			
	$\ln\frac{\%{\mathrm{Stress}}_{\left[26-50\right]}}{\%{\mathrm{Stress}}_{\left[0-25\right]}}$		$\ln\frac{\%{\mathrm{Stress}}_{\left[51-100\right]}}{\%{\mathrm{Stress}}_{\left[0-25\right]}}$	
	EE	PoD	EE	PoD
(Intercept)	−6.18	1.00	−9.06	1.00
Age-30	0.03	0.68	−0.00	0.51
Low alcohol intake	0.58	**0.81**	−0.80	**0.91**
Moderate alcohol intake	1.28	**0.95**	1.73	**1.00**
Worked hours	0.11	**0.93**	0.07	**0.86**
Subjective evening stress	−0.37	**0.98**	−0.21	**0.92**
Subjective evening energy	0.19	**0.86**	0.15	0.56
Active hours	0.09	0.66	0.14	**0.75**
Average daily stress-30	0.06	**0.99**	0.05	**0.98**

EE, estimated effect; PoD, probability of direction. PoD > 0.75, corresponding to Bayes factor > 3 are highlighted in bold.

Except for low alcohol intake, all effects with PoD > 0.75 shared their direction in both medium-to-low and high-to-low ALR comparisons. The low alcohol intake was associated with increased (PoD = 0.81) medium stress and decreased (0.91) high stress. On the other hand, moderate alcohol intake increased both medium and high-stress proportions, as expected from other literature studies (56), evidencing a negative impact of alcohol consumption on nocturnal HRV during the immediate night. The amount of daily work hours was also associated with increased nocturnal stress levels (PoD of 0.93–0.86), which reduced the overnight recovery. Subjective evening stress was negatively associated (PoD of 0.98–0.92) with medium and high-stress levels measured by the smartwatch. Despite being counterintuitive, this may reflect a dissociation between perceived emotional stress and physiological responses, as smartwatch measurements rely on HRV, which may not always align with emotional self-assessments (57). Subjective evening energy was positively associated with medium stress levels (PoD = 0.86). In addition, a substantial increase in daily active hours may reduce heart rate variability (HRV), leading to elevated nocturnal stress. Specifically, daily active hours were positively associated with high nocturnal stress (PoD = 0.75), likely due to their impact on decreasing HRV. Finally, both medium and high levels of nocturnal stress were strongly and consistently associated with average daily stress (PoD = 0.99–0.98), indicating that stress experienced during the day is also mirrored during sleep. Estimated sigmas for random intercepts, quantifying individual-level variability, in modeling the two ALR-transformed outcomes (medium, and high nocturnal stress to low nocturnal stress) were 2.64 and 2.29, respectively, due to zero-inflated amount of time in the medium and high nocturnal stress. Visual analysis of residual plots did not reveal any strong violations of homoscedasticity assumption; however, further analysis is needed to identify possible omitted predictors.

## 4 Discussion

This study exploits data collected through wearable devices to examine sleep stage proportions and nocturnal stress as markers of coping capacity in the context of burnout prevention. As PPG-derived HRV parameters were found to be sufficient in several clinical monitoring studies (58, 59), but not perfectly accurate, we decided to evaluate daily-level aggregates, instead of the signals themselves, as the wearable-derived behavioral trajectories are informative on an individual level (20). In the latest few years, researchers investigated the relationships between physiological data collected using wearable devices and burnout. The “BROWNIE” study (NCT05481138), involving more than 300 hospital nurses, aims at developing AI algorithms and dynamic Bayesian Networks for burnout prevention through continuous wearable data (60). In the “SMART-R” study, authors explored the relationship between sleep and activity data in a cohort of medical residents and found no significant differences in sleep and activity data aggregated over 30 and 90 days between individuals who exhibited burnout and those who did not (61). Instead, in the “Intern Health Study,” the authors found that physical activity and sleep behaviors are indicators of stress resilience (62). Additional references on the use of wearable technologies for burnout detection can be found in a recent review (63). In this initial study, we applied a Bayesian mixed-effects model to longitudinal data to assess individual variability and the shared effects of behaviors and lifestyles on interdependent compositional sleep and nocturnal stress profiles. Despite a relatively small sample, the Bayesian approach provided insights into estimating both the magnitude and direction of effects. The results of this analysis showed that increased daily stress correlated with higher nocturnal stress and reduced deep sleep, while active hours promoted deeper, more restorative sleep. Furthermore, alcohol negatively affected REM sleep, particularly at moderate intake. These findings aligned with existing literature and demonstrate the feasibility of wearables in tracking clinically relevant relationships. All results should be interpreted as preliminary, given the small sample size, short measurement window, absence of participants diagnosed with burnout in the study population, and limited diversity of the study population. Rather than informing health policy, these findings serve as a proof of concept for the feasibility of using aggregated values derived from potentially noisy wearable signals. Despite possible inaccuracies in the raw measurements, daily-level aggregates—modeled while accounting for individual variability through random intercepts—provided meaningful insights into participants’ behavioral patterns, as the authors also discussed in the previous work (64).

Based on a non-burnout population, the study provided insights into the potential for long-term monitoring with commercial wearables. It also provided insights for designing future studies and systems for lifestyle adjustments and burnout prevention. The observed interactions between alcohol, stress, and REM sleep reinforced the role of restorative sleep in cognitive recovery and highlighted the adverse effects of stress on sleep quality. In addition, a mismatch between physiological (HRV-based) and emotional (self-reported) stress highlighted the complexity of stress perception. Identifying key physiological indicators linked to coping capability could support the development of adaptive interventions. Our future research will focus on expanding the participant pool in terms of size and diversity, as the sample size in this study is a limitation, to validate and generalize the findings. With greater sample sizes and longer observation windows, the aim will be to also reconsider adding more covariates and possibly incorporate non-linear functions for enhancing personalized biomarker discovery and informing systems aiming to improve sleep, resilience, and overall well-being.

The preliminary findings of this analysis contribute to the growing body of research exploring how daily physiological and behavioral data can be exploited for understanding of the individual-level metrics on coping capacity and subsequent informing of burnout prevention strategies. By exploring associations between specific lifestyle factors, such as alcohol intake, activity levels, and stress, that influence key markers of coping capacity (i.e., nocturnal HRV and sleep stage proportions), this study highlights that continuous monitoring through wearables can support early identification of burnout-related dysregulation. These insights can inform AI-enhanced solutions for managing stress and promoting mental well-being through targeted personalized adaptive interventions. Importantly, our interpretable modeling approach complements recent work on AI applications in mental health, which increasingly leverage physiological and behavioral data to adaptively support users at risk.

## Data Availability

The datasets presented in this article are not readily available due to participant privacy. Requests to access the datasets should be directed to the corresponding author.

## Footnotes

^1^ https://developer.garmin.com/gc-developer-program/health-api/
